# Data identification of determinants affecting the adoption of sustainable construction: The perspective of residential building developers

**DOI:** 10.1016/j.dib.2021.107556

**Published:** 2021-11-07

**Authors:** Noorsaidi Mahat, Joseph Tah, Christos Vidalakis, Achara Khamaksorn, Orawit Thinnukool, Mohd Iskandar Abd Malek

**Affiliations:** a Faculty of Technology, Design and Environment, School of the Built Environment, Oxford Brookes University, Oxford, UK; b Faculty of Architecture, Planning and Surveying, Universiti Teknologi MARA, Cawangan Sarawak, Malaysia; c College of Arts, Media, and Technology, Chiang Mai University, Chiang Mai, Thailand; d Faculty of Engineering and Built Environment, Universiti Kebangsaan Malaysia, Malaysia

**Keywords:** Likert scale, Questionnaire, Sustainable construction, Barriers, External drivers, Adoption

## Abstract

This data paper shows the perspectives by developers of housing on factors of the sustainable construction adoption. The Data was collected by using formal Likert scale questionnaire as main instrument. Simple random sampling was used to assign questionnaires to respondents. Data samples were evaluated using index and rating. The data will provide information on the most variables that considered as main factors for sustainable construction adoption. The importance between variables can provide information that will contribute to the expedition of sustainable practise in housing projects in Malaysia.

## Specifications Table

**Table:** 

Subject	Building Construction (BC)
Specific subject area	Sustainable Construction (SC)
Term of Definition	Sustainable Construction (SC); *application, practices, innovation, technologies, products, approaches, measures, initiatives, processes and technical means to be employed to accomplish the aim of “energy saving, water saving, material saving, land saving, and environmental protection” during the whole life cycle of the construction*
Type of data	Table Text file
How data were acquired	Field survey
Data format	Raw, filtered and analyzed data
Parameters for data collection	Simple percentages and severity index were used as analytical tool of the generated data. SPSS (Statistical Packages for Social Science Students) was used in determining the nature, strength and pattern of relationships among the cost determinants and variables. The factors were ranked in order of their degree of severity
Description of data collection	Structured questionnaires involving closed-ended queries were distributed to developers belonging to the REHDA (Real Estate and Residential Building Developers ‘Association Malaysia) within 365 registered developers’ company. The 365 questionnaires were distributed via emails to the registered developers. A total of 103 responses were received of which 101 were deemed valid for analysis after data screening, thus representing 28% response rate.
Data source location	Klang Valley, Malaysia
Data accessibility	The article is in public repository https://www-scopus-com.ezaccess.library.uitm.edu.my/record/

## Value of the Data

• The data is useful in research that involves studying sustainable construction adoption.
• Data presented is useful in studying factors affecting sustainable construction adoption that would help client and professional in construction project.
• The data is valuable to construction project professionals and could be used in policy formulation.
• The data could be used as basis of comparison with that of other countries in terms of sustainable construction.

## 1 Data Description

Malaysian developers are known to lack environmental knowledge as a developing country and lack green strategic priorities [1]. There is no literature on the extent of market conduct of developed countries. This paper brings the developers, who are essential decision makers to lead the phase of sustainable development, as an object of study, into addition and completion of the related research. Based on the consideration of the individual factors of the developers [2,3], this research tend to have perception from them in order to obtain the information. The significant barriers of Sustainable Construction (SC) adoption that were identified in literature were listed on the questionnaire and respondents were asked to rank them on a scale of 1 to 5. The findings of the responds are presented in Table 1. It was observed that the most ranked barriers by developers is ‘high initial cost and investment’. This recorded a mean of 4.25. Table 2 shows the responses from the various respondents interviewed. The results show that ‘enhance public image and competitiveness in the industry’, ‘Improve organisation's standard and reputation’ and ‘increase value or premium of the property’ are the key benefits associated with the adoption of SC. The significant drivers of sustainable construction adoption that were identified in literature were listed on the questionnaire and respondents were asked to rank them on a scale of 1 to 5. The findings of the responds are presented in Table 3. It was observed that the most ranked driver by developers is ‘financial support (Incentives/tax rebates/subsidies, high profit margin)’. This recorded a mean of 4.28. The questionnaire is provided as a supplementary file.

**Table 1:** Ranking of perceived barriers of sustainable construction adoption.

No.	Perceived Barriers of Sustainable Construction Adoption	Mean	Standard Deviation	Ranking
1	High initial cost and investment	4.25	0.684	1
2	Insufficient initiatives & support by government in term of tax rebates/subsidies/incentives	4.22	0.743	2
3	Lack of improvement of legislation, Building code and byelaws	4.16	0.689	3
4	Lack of skilled tradesman for sustainable construction	4.14	0.749	4
5	Overlapping of roles among the government agencies	4.14	0.749	5
6	Lack of project team commitment	4.08	0.796	6
7	Insufficient skills about sustainability	4.08	0.794	7
8	Lack of consideration of supplier and manufacturer	4.07	0.778	8
9	Lack of buyer demand and understanding of sustainability	4.03	0.806	9
10	Lack of understanding of cost vs benefits in term of sustainable implementation	4.02	0.836	10

**Table 2:** Ranking of perceived benefits of sustainable construction adoption.

No.	Perceived Benefits of Sustainable Construction Adoption	Mean	Standard Deviation	Ranking
1	Enhance public image and competitiveness in the industry	4.43	0.589	1
2	Improve organisation's standard and reputation	4.36	0.593	2
3	Increase value or premium of the property	4.16	0.689	3
4	Consume less energy	4.03	0.780	4
5	Maximise recycling and reduction of waste production	4.01	0.806	5
6	Increase returns and cost savings	3.99	0.831	6
7	Reduce construction costs	3.97	0.830	7
8	Reduce operation and maintenance costs	3.97	0.832	8
9	Improve indoor environmental quality	3.97	0.890	9
10	Improve occupant productivity	3.93	0.725	10

**Table 3:** Ranking of external drivers of sustainable construction adoption.

No.	Perceived External Drivers of Sustainable Construction Adoption	Mean	Standard Deviation	Ranking
1	Financial support (Incentives/tax rebates/subsidies, high profit margin)	4.28	0.665	1
2	Legislative and building regulation	4.18	0.669	2
3	Availability of rating system. E.g. Green Building Index (GBI)	4.03	0.793	3
4	Awareness and knowledge by top management	4.09	0.763	4
5	Availability of new and integrated technology and material	4.02	0.812	5
6	Availability and access to green products, materials and technology	3.99	0.818	6
7	Cost efficiency and risk	3.98	0.824	7
8	Availability of sustainable construction research funding	3.97	0.830	8
9	Client/buyer awareness and demand	3.96	0.734	9
10	Availability and supports of local suppliers and manufacturers	3.93	0.863	10

## 2 Experimental Design, Materials and Methods

The study aimed to explore the perspectives of housing developers on sustainable construction adoption. To attain these objectives, structured questionnaires involving closed-ended queries were distributed to developers belonging to the REHDA (Real Estate and Residential Building Developers ‘Association Malaysia) within 365 registered developers’ company. The 365 questionnaires have been sent to the registered developers via email. A total of 103 responses were received of which 101 were deemed valid for analysis after data screening, thus representing 28% response rate. Survey data is evaluated according to percentages and mean ratings. The questionnaire designed to recognise advantages, challenges and drivers to sustainable construction and the respondents measured how each barrier and driver has a 5-point Likert scale to impact sustainable construction. Here respondents were asked to indicate their degree of agreement with the barriers on the Likert scale of 5 = strongly agree, 4 = agree, 3 = fairly agree (average), 2 = disagree, 1 = strongly disagree. A factor with a mean score of 2.5 and above was considered significant for the purposes of this study.

## Ethics Statement

Consent was obtained from university research ethics committee (UREC No 161068) for experimentation with human subjects. The privacy rights of human subjects are always be observed.

## Declaration of Competing Interest

The authors announce that there are no conflicting financial interests or personal relationships, known or unknown, that are important to the work reported in this paper, that could be considered a conflict of interest.

## Appendix Supplementary materials

Image, application 1

Image, application 2

Image, application 3
