# Supplementary material for: Data identification of determinants affecting the adoption of sustainable construction: The perspective of residential building developers
Source: Data Brief. 2021 Nov 7;39:107556. doi: 10.1016/j.dib.2021.107556 (PMC8603009; doi:10.1016/j.dib.2021.107556)
Supplement: Supplementary file 1 [file mmc1.pdf]

OXFORD BROOKES UNIVERSITY

QUESTIONNAIRE TO HOUSING COMPANIES

Sustainable Construction Adoption in Residential Building Projects: A Case of Residential Building Developers in Malaysia

This study aims to investigate the factors affecting sustainable construction adoption in residential building projects in Malaysia with a particular focus on residential building developers. The purpose of this survey is to:

- Investigate the level of awareness and knowledge in sustainable construction
- Identify the importance of factors affecting sustainable construction adoption by residential building developers.

The survey takes approximately 20 minutes to complete. If possible, please answer all questions in one session. Your answers will be kept strictly confidential and anonymous and will be used for academic purpose only.

Appendix A: Interviewer Administered Questionnaire

SECTION 1: Demographic information

a. Your position in the project

**Position**	
Managing Director	
Manager	
Executive	
Others.Please specify……………	

b. Years of experience in construction industry

**Years of experience**	
Less than 5 years	
5 to 10 years	
More than 10 years	

SECTION 2: Project Details

c. What type(s) of residential building(s) have you been involved with?

**Type of residential building**	
Terraced	
Bungalow	
Semi-D	
Apartments/Flats	
Town Houses	
Condominium	

d. What is the size of your company?

**Employees**	
Less than 20 (Small Business)	
21-50 (Medium Business)	
51 or more (Large Business)	

OXFORD BROOKES UNIVERSITY

SECTION 3: **Awareness and Knowledge**

1. What is your level of familiarity with the following Sustainable Construction areas in the residential building projects? Please **rate** (from **1-5, not at all to very great**) the following based on your awareness?

**Sustainable construction area**	**None**	**Basic**	**Good**	**Very good**	**Excellent**
**Environment**<br>- Reducing greenhouse emissions and pollution levels, Managing natural resources, Waste Management.<br>e.g. Selection of sustainable materials, Sustainable site practices, Waste reduction and disposal, Green design.	1	2	3	4	5
**Economic**<br>- Internalise cost, Value for money.<br>e.g. Green purchasing, purchase value for money.	1	2	3	4	5
**Social**<br>- Promote public participation, Provide high customer satisfaction.<br>e.g. Receiving positive feedback from community, improving of building image and reputation.	1	2	3	4	5

2. How often do you use the following Sustainable Construction practices in the residential building projects you are involved in?

**Environment**	**Never**	**Not often**	**Less often**	**Very often**	**Alwa ys**
Waste reduction & disposal on site	1	2	3	4	5
Improve building design in term of energy efficiency	1	2	3	4	5
Improve building design in term of Air indoor environmental Quality Index	1	2	3	4	5
Improve building design in term of water efficiency and conservation	1	2	3	4	5
Manage resources efficiently	1	2	3	4	5
Use of site landscaping	1	2	3	4	5
Minimise potential environmental pollution risks	1	2	3	4	5
use materials based on eco-labelling guideline	1	2	3	4	5
Use of off-site Construction; the Industrialised Building system (IBS)	1	2	3	4	5
Use of Building Integrated Management (BIM)	1	2	3	4	5
Others. Please specify………………	1	2	3	4	5
**Economic**	**Never**	**Not often**	**Less often**	**Very often**	**Alwa ys**
Purchase to meet minimum environmental criteria	1	2	3	4	5
Purchase value for money	1	2	3	4	5
Use tender selection based on most economically advantageous tender	1	2	3	4	5
Use mechanism to avoid fines for	1	2	3	4	5

environmental destruction					
Use E-tendering	1	2	3	4	5
Giving priority to suppliers who promotes efficient waste management with long term policy	1	2	3	4	5
Others. Please specify………………	1	2	3	4	5
**Social**	**Never**	**Not often**	**Less often**	**Very often**	**Alwa ys**
Create awareness among project team of environmentally-friendly culture	1	2	3	4	5
Receive positive feedback from community	1	2	3	4	5
Enhance environmentally friendly culture	1	2	3	4	5
Produce educated stakeholders in sustainable project	1	2	3	4	5
Gaining business satisfaction in terms of good image/reputation	1	2	3	4	5
Use of policy at project level urging environmental awareness	1	2	3	4	5
Have an agreed definition of "green" by project team	1	2	3	4	5
Other. Please specify………………	1	2	3	4	5

SECTION 4: Factors affecting the adoption of Sustainable Construction in residential building projects in Malaysia

3. To what extent do you agree (or disagree) with the following statements?

**Statement (attitude)**	Strongly disagree	Disagree	Neither disagree nor agree	Agree	Strongly agree
Sustainable construction practices are not economically viable or achievable when utilised in residential buildings	1	2	3	4	5
Sustainable construction practices in comparison with traditional construction practices are more difficult to apply in residential buildings	1	2	3	4	5
Traditional construction practices in comparison with Sustainable construction practices have more environmental, economical and social advantages	1	2	3	4	5
**Statement (Subjective norms)**	Strongly disagree	Disagree	Neither disagree nor agree	Agree	Strongly agree
I believe there is pressure from my senior management to adopt Sustainable construction practices in residential building projects	1	2	3	4	5

My senior management think that I should not adopt Sustainable construction practices concept in residential building projects	1	2	3	4	5
My senior management would approve of me adopting Sustainable construction practices in residential building projects	1	2	3	4	5
My senior management would support me adopting Sustainable construction practices in residential building projects	1	2	3	4	5
**Statement (Perceived behavioural control)**	Strongly disagree	Disagree	Neither disagree nor agree	Agree	Strongly agree
The decision whether or not to adopt Sustainable Construction practices is entirely up to me	1	2	3	4	5
I could adopt Sustainable Construction practices if I wanted to	1	2	3	4	5
I have plenty of opportunities to adopt Sustainable Construction practices	1	2	3	4	5
Adopt Sustainable Construction practices is inconvenient	1	2	3	4	5
Adopt Sustainable Construction practices is a hassle	1	2	3	4	5
I know what practices of Sustainable Construction can be adopted	1	2	3	4	5

4. How important could the following factors be in motivating you or driving your organisation to adopt Sustainable Construction practices in residential buildings in Malaysia?

**Relative Advantage (Perceived Benefits)**	Not important	Less important	Neutral	Important	Very important
Improvement of energy efficiency	1	2	3	4	5
Improvement of water efficiency	1	2	3	4	5
Minimisation of health and safety risks	1	2	3	4	5
Reduction of environmental impact during construction	1	2	3	4	5
Reduction of construction waste during construction	1	2	3	4	5
Maximisation of recycling and reduction of waste production	1	2	3	4	5
Achievement of high building quality	1	2	3	4	5
Control of working conditions and reduction of local impacts	1	2	3	4	5

Improvement of indoor environmental quality	1	2	3	4	5
Improvement of occupant productivity	1	2	3	4	5
Compensation for the shortage of skilled workers	1	2	3	4	5
Consumption of less energy	1	2	3	4	5
**Image**	Not important	Less important	Neutral	Important	Very important
Improvement of building image	1	2	3	4	5
Improvement of organisation's standard and reputation	1	2	3	4	5
Expansion of markets shape	1	2	3	4	5
Increased value or premium of the property	1	2	3	4	5
**Government**	Not important	Less important	Neutral	Important	Very important
Adequate support from Government (government policies)	1	2	3	4	5
**Cost**	Not important	Less important	Neutral	Important	Very important
Reduction of operation and maintenance costs	1	2	3	4	5
Increased returns and cost savings	1	2	3	4	5
Reduction of construction costs	1	2	3	4	5
Other reasons. Please specify	Not important	Less important	Neutral	Important	Very important
	1	2	3	4	5
	1	2	3	4	5
	1	2	3	4	5

5. How important could the following factors be in demotivating you or restraining your organisation to adopt Sustainable Construction practices in residential buildings in Malaysia?

**Lack of ease of use**	Not important	Less important	Neutral	Important	Very important
Time constrains	1	2	3	4	5
Contract requirement/procurement practices	1	2	3	4	5
Limited company policies	1	2	3	4	5
**Trialability**	Not important	Less important	Neutral	Important	Very important
Inability of sustainable construction technology advance testing	1	2	3	4	5
**Compatibility**	Not important	Less important	Neutral	Important	Very important
Sustainable construction technology is not suitable to the nature of construction	1	2	3	4	5
Limited local sustainable materials	1	2	3	4	5

**Avoidance**	Not important	Less important	Neutral	Important	Very important
Lack of consideration of client	1	2	3	4	5
Lack of consideration of supplier and manufacturer	1	2	3	4	5
Resistance to change from traditional ways of doing work	1	2	3	4	5
Lack of client demand and understanding of sustainability	1	2	3	4	5
**Complexity**	Not important	Less important	Neutral	Important	Very important
Sustainable construction complexity (complicated technology and not easy to adopted)	1	2	3	4	5
**Cost**	Not important	Less important	Neutral	Important	Very important
Increase in construction cost	1	2	3	4	5
Financial resources to address sustainability	1	2	3	4	5
Risk of unforeseen cost due to higher initial investment cost	1	2	3	4	5
**Knowledge/Awareness**	Not important	Less important	Neutral	Important	Very important
Lack of education of benefits and incentives on sustainable buildings	1	2	3	4	5
Lack of understanding of environmental implications and its solution	1	2	3	4	5
Lack of understanding of cost vs. benefits in term of sustainable implementation	1	2	3	4	5
Lack of public awareness understanding sustainability	1	2	3	4	5
Lack of awareness and knowledge among project team and contractors on sustainability	1	2	3	4	5
**Training**	Not important	Less important	Neutral	Important	Very important
Insufficient skills about sustainability	1	2	3	4	5
Lack of skilled tradesman for sustainable construction	1	2	3	4	5
Lack of training of benefits and incentives on sustainable buildings	1	2	3	4	5
**Government**	Not important	Less important	Neutral	Important	Very important
Overlapping of roles among the government agencies	1	2	3	4	5
Slow government programs about sustainability	1	2	3	4	5
Insufficient initiatives & support by government	1	2	3	4	5
**Observability**	Not important	Less important	Neutral	Important	Very important
Lack of research and innovation about sustainability	1	2	3	4	5
Other reasons: please specify					
	1	2	3	4	5
	1	2	3	4	5
	1	2	3	4	5

SECTION 5: Potential for the adoption of Sustainable Construction practices in residential building projects in Malaysia

6. To what extent do you agree (or disagree) with the following statement?

**Statement**	Strongly Disagree	Disagree	Neither Disagree nor Agree	Agree	Strongly Agree
Sustainable residential buildings have the potential to become a dominant practice in Malaysia	1	2	3	4	5

7. To what extent do you agree (or disagree) with the following statements indicating reasons to adopt Sustainable Construction in residential building in Malaysia?

**Relative Advantage**	Strongly Disagree	Disagree	Neither Disagree nor Agree	Agree	Strongly Agree
Special features of sustainable residential building will attract more buyers	1	2	3	4	5
Sustainability is new way of life, thus, can tap into new market and increasing demand	1	2	3	4	5
Enhance competitiveness in the industry and to mark achievement as part of company performance	1	2	3	4	5
Demand for sustainable residential building is increasing	1	2	3	4	5
Venturing into sustainable construction ensures more opportunities for future projects	1	2	3	4	5
Sustainable residential building is a distinct product in the market, can be great marketing tool	1	2	3	4	5
Improvement in employee productivity reduces absenteeism and building-related health problems due to healthy indoor environment	1	2	3	4	5
Sustainable construction fulfils the social needs by concerning on community needs and social risk, thus projecting developers who care about its society	1	2	3	4	5
answer toward avoiding harmful activity toward the environment as it based on ecologically sound principles	1	2	3	4	5
Sustainable construction as a safe way to avoid any risk of noncompliance in development.	1	2	3	4	5
**Image**	Strongly Disagree	Disagree	Neither Disagree nor Agree	Agree	Strongly Agree
Enhance public image and competitiveness in the industry	1	2	3	4	5
As proof that developers have met environmental standards and performance	1	2	3	4	5
**Government**	Strongly Disagree	Disagree	Neither Disagree nor Agree	Agree	Strongly Agree
Financial incentives can be obtained through tax exemption, fee waiver, and loans from the	1	2	3	4	5

government.					
**Cost**	Strongly Disagree	Disagree	Neither Disagree nor Agree	Agree	Strongly Agree
sustainable residential building has high potential regarding cost-saving in long term	1	2	3	4	5
An increase in capital cost is marginal when the concept is being introduced early in the project life	1	2	3	4	5
Developers/ occupiers can enjoy cost savings during operational and maintenance	1	2	3	4	5
Others: please specify	1	2	3	4	5
	1	2	3	4	5
	1	2	3	4	5
	1	2	3	4	5

8. To what extent do you agree (or disagree) that the following are important reasons why Sustainable Construction is not adopted in residential building projects in Malaysia.

**Lack of Ease of Use (Perceived Barriers)**	Strongly Disagree	Disagree	Neither Disagree nor Agree	Agree	Strongly Agree
Lack of cooperation between project stakeholders	1	2	3	4	5
Time constrains	1	2	3	4	5
Contract requirement/procurement practices	1	2	3	4	5
Limited company policies	1	2	3	4	5
**Trialability**	Strongly Disagree	Disagree	Neither Disagree nor Agree	Agree	Strongly Agree
Inability of sustainable construction technology advance testing	1	2	3	4	5
**Compatibility**	Strongly Disagree	Disagree	Neither Disagree nor Agree	Agree	Strongly Agree
Sustainable construction technology is not suitable to the nature of construction	1	2	3	4	5
Limited local sustainable materials	1	2	3	4	5
**Avoidance**	Strongly Disagree	Disagree	Neither Disagree nor Agree	Agree	Strongly Agree
Lack of consideration of client and stakeholder	1	2	3	4	5
Resistance to change from traditional ways of doing work	1	2	3	4	5
Lack of client demand and understanding of sustainability	1	2	3	4	5
**Complexity**	Strongly Disagree	Disagree	Neither Disagree nor Agree	Agree	Strongly Agree
Sustainable construction complexity (complicated technology and not easy to adopted)	1	2	3	4	5
**Cost**	Strongly Disagree	Disagree	Neither Disagree nor Agree	Agree	Strongly Agree
Compared to traditional construction projects, more initial investment is generally needed for	1	2	3	4	5

sustainable construction					
**Knowledge/Awareness**	Strongly Disagree	Disagree	Neither Disagree nor Agree	Agree	Strongly Agree
Low level consciousness of sustainable construction caused by a lack of related information and knowledge	1	2	3	4	5
**Training**	Strongly Disagree	Disagree	Neither Disagree nor Agree	Agree	Strongly Agree
Lack of skilled and qualified workers to the adoption of related technologies and methods	1	2	3	4	5
**Government**	Strongly Disagree	Disagree	Neither Disagree nor Agree	Agree	Strongly Agree
Insufficient and ineffectiveness of government policies and regulations mitigating the negative impact of construction activities on the environment and society.	1	2	3	4	5
**Observability**	Strongly Disagree	Disagree	Neither Disagree nor Agree	Agree	Strongly Agree
Lack of research and innovation about sustainability	1	2	3	4	5
Other: Please specify	Strongly Disagree	Disagree	Neither Disagree nor Agree	Agree	Strongly Agree
	1	2	3	4	5
	1	2	3	4	5
	1	2	3	4	5

9. What are your suggestions to adopt successful Sustainable Construction in residential building projects in general?
……
……
……
……

10. This survey will be followed by a series of interviews looking at particular themes that might emerge from this survey. Please provide your details below if you are happy to participate in an interview?

Name: ……………………………………………………….
Company: ………………………………………………….
E-mail Address: ……………………………………………
Telephone number: ………………………………………..

THANK YOU FOR YOUR CO-OPERATION

Noorsaidi Mahat
PhD Research Student
School of Built Environment, Faculty of Technology,
Design and Environment, Oxford Brookes University,
Oxford, UK.
Email: 15110098@brookes.ac.uk
Tel: +60194991770 (Malaysia) / +447533499939 (UK)
